# Learning management system effectiveness and trainee perceptions

**DOI:** 10.1186/s12909-026-09535-7

**Published:** 2026-06-04

**Authors:** Noha Sobhy Abdeen, Nayera Samy Mostafa, Dina Shawky Alzifzaf

**Affiliations:** 1 Medical Education Department, Faculty of Medicine, Ain Shams University, Cairo, Egypt; 2 Community, Environmental and Occupational Medicine Department, Faculty of Medicine, Ain Shams University, Cairo, Egypt

**Keywords:** E-Learning, Learning Management System Platform, Egyptian Fellowship Training, Improvement

## Abstract

**Background:**

Despite the increasing integration of digital learning in postgraduate medical education, the utilization of Learning Management Systems (LMS) among Egyptian fellowship trainees remains suboptimal. Limited engagement and unclear perceived value highlight the need for structured training interventions. We aimed to assess the effectiveness of e-learning and the use of the LMS platform in enhancing hybrid training methods for Egyptian fellowship trainees at El-Behera governorate training centers.

**Methods:**

A quasi-experimental pre–post interventional study was conducted on 211 newly enrolled Egyptian fellowship trainees at El-Behera training centers between August and December 2024. Participants completed a validated self-administered questionnaire assessing perceptions of e-learning and LMS use. An orientation-based training intervention was delivered, including hands-on LMS navigation and resource utilization. Paired analysis was performed on 111 participants who completed both pre- and post-intervention assessments.

**Results:**

Post-intervention, participants demonstrated significant improvements in perceived institutional support and overall perception of distance learning. Significant positive changes were observed across all LMS domains, including usability, accessibility of resources, perceived impact on clinical development, personalization of learning, and course scheduling (all *p* < .01). Additionally, the proportion of trainees who believed that LMS facilitates achievement of training requirements increased markedly (61.3% vs. 87.4%). However, no significant changes were observed in engagement levels or perceived effectiveness of distance learning.

**Conclusions:**

Structured LMS training significantly improves trainee perceptions and perceived utility of digital learning platforms but has limited impact on engagement and contextual barriers. Institutions should prioritize continuous training, infrastructure development, and interactive LMS design to maximize educational outcomes in hybrid postgraduate training models.

**Supplementary Information:**

The online version contains supplementary material available at 10.1186/s12909-026-09535-7.

## Introduction

Clinical guidelines and health care delivery competencies are changed and updated very fast. Medical residents “adult learners”, during their postgraduate training and studies should be eager to look up these updates to increase their clinical skills and reasoning with patients. They should be self-directed to tailor their learning plan and achieve relevant skills and competencies in their career path [1].

Egyptian fellowship began its specialized training activities as a postgraduate professional certificate for health care workers under the supervision of the Supreme Committee for Medical Specialties. The training is conducted at accredited hospitals and primary health care centers in the form of on-job training activities, in its first year the training is mostly directed to basic foundation skills of each specialty, followed by a written first part exam then, advanced skills subsequently introduced in the remaining training years, and final second part written exam and third part clinical exam are required for graduation. The training is mostly learner centered, while the training progress to be supervised by hospital trainers’ then regular monthly meeting with the scientific supervisor held either physically or online meetings to monitor candidate progress [2].

As a result of COVID19 pandemic and its subsequent effect on medical education all over the world, that hinders most traditional physical training and class activities, the Egyptian fellowship supreme committee adopts its own learning management system (LMS) platform to overcome this challenge and to offer the trainees and the trainers a plenty of medical libraries (Incision academy – Elsevier – clinical key –ACLAND anatomy –Medline –Egyptian knowledge bank), also LMS electronic management department created specialty playlists, study guides, national clinical guidelines, training logbook and portfolio. All board scientific councils were oriented how to friendly use it to schedule the training effectively [3].

With the foundation of the Egyptian health council in 2023 that have supervised all training activities of the Egyptian fellowship, the concept of online logbook and portfolio registration and tracing trainee achievement has been adopted. According to training bylaw, each trainee should attach training and exam pre-requisites documents to be reviewed by the scientific council of the specialty including (logbook - portfolio - professional progress plan –certificates of foundation courses, workshops, conferences- continuing medical education- scientific meeting participation [4]. Even though it is proved that trainees who integrate E-learning and physical training activities will achieve clinical skills more effectively than trainees who adopt only physical training activities [5], most of Egyptian fellowship trainees did not integrate E-learning to their portfolio achievements and did not access LMS medical libraries as well.

Primary Aim was to evaluate the effect of an LMS-based orientation intervention on Egyptian fellowship trainees’ perceptions of e-learning. Secondary Objectives were to assess trainees’ perceived challenges related to LMS use, to evaluate changes in usability, satisfaction, and perceived educational value, and to identify trainee and trainer recommendations for improving LMS implementation.

## Participants and methods

This quasi-experimental pre–post educational interventional study was carried out on 211 fresh joined Egyptian fellowship trainees, at El-behera training centers – Egyptian fellowship training programs aged from 25 to 40 years old, and both sexes.

A quasi-experimental design without a control group was adopted due to the institutional requirement to provide equal training exposure to all newly enrolled trainees, which limited the feasibility of randomization.

Only participants who completed both pre- and post-orientation questionnaires (*n* = 111) were included in the paired comparative analysis.

An informed written consent was obtained from the participants. The study was done after approval from the Ethical Committee faculty of medicine- Ain Shams University (IRB No: FMASU MS462/2024) from August 2024 to December 2024.

Participant recruitment was held in the monthly scientific meetings of all specialities, and the researcher explained its rationale to the candidates.

Data collection was carried out during the scientific meetings where trainees were asked to self-evaluate their knowledge and self-perceptions in E-learning and platform approaches through online predesigned questionnaire that included a five-point Likert-type scale items, dichotomous items, and direct selection items.

### Questionnaire was subdivided into four categories

1. Introductory items about trainees’ socio-demographic data, interests and their preferred teaching and learning approach [6].
2. Questions linked to E-learning and online platforms [7].
3. Questions linked to the candidate attributes build out during the on-job daily practices [8].
4. Open ended questions about recommendations for the future.

The questionnaire was validated by expert opinions in medical education department, faculty of medicine –Ain Shams University. The overall internal consistency of the instrument, as measured by Cronbach’s Alpha, is 0.806, based on 10 items, indicating acceptable reliability.

Then, split half reliability was done by dividing the questions into two parts:

- Part 1, comprising 5 items related to perceptions of university support and effectiveness of distance learning, has a Cronbach’s Alpha of 0.753.
- Part 2, consisting of 5 items focused on the usability and resource availability of the Egyptian Fellowship LMS has a Cronbach’s Alpha of 0.877.

The questionnaire was developed based on prior literature and aligned with study objectives. Content validity was established through review by three experts in medical education and digital learning. A pilot study was conducted on 20 trainees to ensure clarity and feasibility, and necessary modifications were applied.

The questionnaire was originally developed in English and translated into Arabic using forward–backward translation to ensure linguistic validity as Supplementary File 1.

Although the Spearman-Brown coefficient was relatively low (0.417), the overall Cronbach’s alpha (0.806) indicated acceptable internal consistency. The discrepancy may be attributed to the small number of items within each split-half.

**Table:** 

Overall Cronbach’s Alpha (for the 10 items)		Value	0.806
Cronbach’s Alphaf	Part 1	Value	0.753
		N of Items	5^a^
	Part 2	Value	0.877
		N of Items	5^b^
Spearman-Brown Coefficient	Equal Length		0.417

#### Part 1 (a) items

1. How helpful has your university been in providing resources for remote learning?
2. How helpful are your trainers during online study?
3. How effective has distance learning been for you?
4. How engaged do you feel with the online training process?
5. What is your overall perception of distance learning?

#### Part 2 (b) items

1. The Egyptian Fellowship LMS interface is user-friendly, and I can easily access its libraries and playlists.
2. The LMS provides comprehensive resources and materials relevant to my specialty (knowledge, skills, attitude).
3. The LMS resources have positively influenced my clinical experiences and professional development.
4. The LMS helps me personalize my learning plan and focus on areas of interest.
5. The LMS enables me to schedule my foundational courses effectively.

The intervention consisted of a structured orientation session (2 h) delivered during scheduled scientific meetings to ensure maximum attendance. The session included:

- Introduction to LMS features
- Demonstration of navigation and resource access
- Practical hands-on training
- Guidance on integrating LMS into training requirements

All trainees attended the session, and trainers were invited to participate and provide feedback regarding LMS implementation and potential improvements.

This analysis indicates that the instrument demonstrates satisfactory internal consistency across its components.

An orientation session was held about Egyptian fellowship LMS, the contents covered which included (LMS libraries and resource, its benefit, how to access and how to get LMS user friendly) in addition to hands on and demonstration of LMS access and navigation.

All fellowship trainers were asked to join orientation sessions to assess, their suggestions for improvement regarding Egyptian fellowship LMS platform performance.

Scheduling orientation sessions in the monthly scientific meeting days (to ensure maximum trainees’ attendance), was done after taking the needed approvals.

The Egyptian fellowship governmental office instructor who was attended to report and to coordinate these sessions.

Thus, study participants at the end of first clinical rotation, all trainees were asked to fill the post orientation form through the same online predesigned questionnaire previously used at the beginning of the study.

The post orientation questionnaire was filled by 111 trainees in a response rate of 52.6%.

The researcher was added to the study participant’s social media groups to facilitate, communicate and send the online form to them.

### Statistical analysis

Statistical analysis was done by SPSS v26 (IBM Inc., Armonk, NY, USA). Quantitative variables were presented as mean and standard deviation (SD) and compared between the two groups utilizing paired Student’s t- test. Qualitative variables were presented as frequency and percentage (%) and were analysed utilizing the Chi-square test or Fisher’s exact test when appropriate. Related-samples Marginal Homogeneity test was used to compare qualitative variables before and after training. Normality of quantitative variables was assessed using the Shapiro–Wilk test. Missing data were handled using pairwise deletion. A two tailed P value < 0.05 was considered statistically significant.

## Results

At baseline, trainees demonstrated generally positive attitudes toward e-learning despite notable barriers, including limited formal training, technical constraints, and environmental distractions. Most participants relied on self-directed learning, with smartphones being the primary device used, and a preference for small-group learning approaches was evident.

Table 1 presents the baseline characteristics and e-learning experiences of the study participants. Overall, trainees demonstrated generally positive attitudes toward distance learning despite notable barriers, including limited formal training and environmental distractions. Overall, while trainees reported moderate to high satisfaction with distance learning and perceived trainer support, engagement levels remained variable, and a substantial proportion had not received prior formal training in e-learning platforms. Common barriers included limited access, technological issues, and reduced interaction. Table 1.

**Table 1:** Distribution of participants’ demographic characteristics, online learning experiences, device accessibility, training exposure, engagement, and perceptions toward distance learning at baseline (*N* = 211)

Variable / Question	Category	*N* (%)
Age (years)		31.9 ± 4 (25–40)
Gender	**Male**	86 (40.8%)
	**Female**	125 (59.2%)
Specialty	**Internal medicine specialties**	52 (24.6%)
	**Surgical specialties**	42 (19.9%)
	**Family medicine**	28 (13.3%)
	**Pediatric / Neonatology**	19 (9.0%)
	**Internal medicine subspecialties**	18 (8.5%)
	**Critical / emergency specialties**	18 (8.5%)
	**Surgical subspecialties**	15 (7.1%)
	**Clinical pharmacy**	7 (3.3%)
	**Nursing specialties**	7 (3.3%)
	**Dentist’s specialties**	5 (2.4%)
Location of Work	**Hospital**	101 (47.9%)
	**Institutes**	62 (29.4%)
	**Primary Healthcare Center**	48 (22.7%)
Task Engagement Method	**Small group task**	124 (58.8%)
	**Individual assignment**	72 (34.1%)
	**Large group task**	15 (7.1%)
Access to a Device	**Yes, 100% of time available**	106 (50.2%)
	**Less than 50% of time**	90 (42.7%)
	**No, share with others**	15 (7.1%)
Devices Used (multiple response)	**Smartphone**	152 (72.0%)
	**Laptop**	94 (44.5%)
	**Tablet**	33 (15.6%)
	**Desktop computer**	8 (3.8%)
Experience at Home	**Learning comfortably at own pace**	125 (59.2%)
	**Better with uninterrupted connectivity**	38 (18.0%)
	**Distracted at home**	30 (14.2%)
	**No time due to responsibilities**	10 (4.7%)
	**Situational challenges unsuitable**	8 (3.8%)
Disturbance at Home	**Family/roommate disturb**	138 (65.4%)
	**No disturbance**	73 (34.6%)
Interest / Adoption of Online Learning	**Motivates for advanced courses**	84 (39.8%)
	**Slow devices/internet discouraging**	50 (23.7%)
	**Difficult due to less face-to-face contact**	35 (16.6%)
	**Difficult without guidance**	32 (15.2%)
	**Post queries to class peers**	26 (12.3%)
	**Avoided due to social isolation**	10 (4.7%)
Training in E-Learning Platforms	**No**	144 (68.2%)
	**Yes**	67 (31.8%)
Who Helped in Training	**Self-directed**	128 (60.7%)
	**Trainer**	45 (21.3%)
	**Scientific supervisor**	23 (10.9%)
	**Senior trainee**	15 (7.1%)
Preferred Method to Clear Doubts	**Ask professor during/after session**	113 (53.6%)
	**Use online materials**	72 (34.1%)
	**Ask peers**	26 (12.3%)
Institute Support	**Extremely helpful**	10 (4.7%)
	**Very helpful**	57 (27.0%)
	**Moderately helpful**	76 (36.0%)
	**Slightly helpful**	40 (19.0%)
	**Not at all helpful**	28 (13.3%)
Trainer Helpfulness	**Extremely helpful**	24 (11.4%)
	**Very helpful**	94 (44.5%)
	**Moderately helpful**	72 (34.1%)
	**Slightly helpful**	20 (9.5%)
	**Not at all helpful**	1 (0.5%)
Effectiveness of Distance Learning	**Extremely effective**	24 (11.4%)
	**Very effective**	89 (42.2%)
	**Moderately effective**	82 (38.9%)
	**Slightly effective**	15 (7.1%)
	**Not at all effective**	1 (0.5%)
Engagement in Online Training	**Very engaged**	58 (27.5%)
	**Somewhat engaged**	67 (31.8%)
	**Neutral**	74 (35.1%)
	**Somewhat disengaged**	10 (4.7%)
	**Very disengaged**	2 (0.9%)
Overall Feeling About Distance Learning	**Excellent**	39 (18.5%)
	**Good**	122 (57.8%)
	**Average**	44 (20.9%)
	**Below average**	6 (2.8%)

Data are presented as mean ± SD (Range) or frequency (%)

Table 2 summarizes participants’ baseline perceptions of the Egyptian Fellowship LMS. While the system was generally viewed positively in terms of usability and resource availability, several challenges were identified, particularly lack of training, accessibility issues, and outdated content. The LMS was generally perceived positively at baseline, particularly in terms of usability, resource availability, and contribution to clinical development. However, several challenges were identified, most notably lack of training, accessibility issues, and outdated content. Despite these limitations, more than half of participants believed that the LMS could support achievement of training requirements.

**Table 2:** Distribution of participants’ opinions regarding the Egyptian fellowship LMS at baseline (*N* = 211) and characteristics of participants who completed the post-training questionnaire (*N* = 111)

Variable / Question	Category	*N* (%)
Egyptian fellowship LMS is user-friendly and easy to access	**Strongly agree**	0 (0.0%)
	**Agree**	110 (52.1%)
	**Neutral**	79 (37.4%)
	**Disagree**	18 (8.5%)
	**Strongly disagree**	4 (1.9%)
LMS provides comprehensive specialty resources (knowledge–skills–attitude)	**Strongly agree**	6 (2.8%)
	**Agree**	110 (52.1%)
	**Neutral**	67 (31.8%)
	**Disagree**	23 (10.9%)
	**Strongly disagree**	5 (2.4%)
LMS resources reflect on clinical experience and professional development	**Strongly agree**	4 (1.9%)
	**Agree**	110 (52.1%)
	**Neutral**	75 (35.5%)
	**Disagree**	17 (8.1%)
	**Strongly disagree**	5 (2.4%)
LMS helps personalize learning plans	**Strongly agree**	7 (3.3%)
	**Agree**	99 (46.9%)
	**Neutral**	79 (37.4%)
	**Disagree**	19 (9.0%)
	**Strongly disagree**	7 (3.3%)
LMS enables effective scheduling of foundation courses	**Strongly agree**	8 (3.8%)
	**Agree**	89 (42.2%)
	**Neutral**	82 (38.9%)
	**Disagree**	27 (12.8%)
	**Strongly disagree**	5 (2.4%)
Overall satisfaction with LMS resources	**Strongly satisfied**	3 (1.4%)
	**Satisfied**	109 (51.7%)
	**Neutral**	71 (33.6%)
	**Dissatisfied**	24 (11.4%)
	**Strongly dissatisfied**	4 (1.9%)
Challenges with LMS	**Lack of training**	54 (26.7%)
	**Accessibility issues**	47 (23.3%)
	**Technical issues**	30 (14.9%)
	**Lack of updated resources**	30 (14.9%)
	**Work overload / time**	26 (12.9%)
	**No challenges**	15 (7.4%)
Has LMS increased likelihood of achieving training requirements?	**Yes**	118 (55.9%)
	**No**	19 (9.0%)
	**Maybe**	74 (35.1%)
Greatest LMS impact on clinical practice	**Knowledge**	101 (49.8%)
	**Clinical skills**	60 (29.6%)
	**Foundation courses**	13 (6.4%)
	**Communication with Supreme Committee**	5 (2.5%)
	**Uncertain of value**	24 (11.8%)
Suggestions for improving LMS	**Increase updated clinical resources**	58 (29.0%)
	**Improve accessibility / technical support**	42 (21.0%)
	**More training & LMS orientation**	40 (20.0%)
	**Increase foundation courses**	12 (6.0%)
	**Nothing**	48 (24.0%)
Additional comments / feedback	**More training/orientation**	37 (18.7%)
	**Frequent updates**	15 (7.6%)
	**More clinical courses**	9 (4.5%)
	**Flexible time**	2 (1.0%)
	**Nothing**	135 (68.2%)

POST-TRAINING SAMPLE (*N* = 111)		
Age (years)		31.4 ± 3.5 (25–40)
Gender	**Male**	47 (42.3%)
	**Female**	64 (57.7%)
Specialty Distribution	**Internal medicine specialties**	28 (25.2%)
	**Family medicine**	16 (14.4%)
	**Surgical specialties**	14 (12.6%)
	**Surgical subspecialties**	12 (10.8%)
	**Internal medicine subspecialties**	10 (9.0%)
	**Critical/emergency specialties**	9 (8.1%)
	**Pediatrics/Neonatology**	7 (6.3%)
	**Clinical pharmacy**	6 (5.4%)
	**Dentists’ specialties**	5 (4.5%)
	**Nursing specialties**	4 (3.6%)
Location of Work (Post-training)	**Institute**	62 (55.9%)
	**Primary Healthcare Center**	36 (32.4%)
	**Hospital**	13 (11.7%)

Data are presented as mean ± SD (Range) or frequency (%). *LMS* Learning management system

Several statistically significant improvements were observed after the training intervention. Participants reported significantly greater institutional support for online learning post-training (*p* = .023), and their overall perception of distance learning improved, with a higher percentage rating it as excellent (29.7% post vs. 18.9% pre; *p* = .028). Significant enhancements were also noted in all core LMS-related domains. These included perceived user-friendliness, access to content libraries (*p* < .001), comprehensiveness of LMS resources (*p* < .001), perceived impact on clinical and professional development (*p* < .001), personalization of learning plans (*p* = .001), and the LMS’s role in effectively scheduling foundation courses (*p* < .001). Overall satisfaction with LMS resources increased markedly as well (*p* < .001). Participants were also significantly more likely to believe the LMS increased their likelihood of achieving training requirements post-training (87.4% vs. 61.3%; *p* = .024). Non-significant changes were observed in perceptions of trainer helpfulness, effectiveness of distance learning, engagement in the online training process, and several LMS challenge categories, indicating these areas remained relatively stable before and after training. Although some shifts were seen in challenges and suggested improvements, they did not reach statistical significance. Table 3.

**Table 3:** Comparison of pre- and post-training participants’ perceptions of factors influencing online learning and their opinions of the Egyptian fellowship Learning Management System (LMS) (*N* = 111)

Variable / Question	Category	Pre *N* (%)	Post *N* (%)	*P*-value
How helpful has your Institute been in offering resources for learning at home?	**Extremely helpful**	4 (3.6%)	13 (11.7%)	0.023*
	**Very helpful**	31 (27.9%)	26 (23.4%)	
	**Moderately helpful**	44 (39.6%)	44 (39.6%)	
	**Slightly helpful**	18 (16.2%)	28 (25.2%)	
	**Not at all helpful**	14 (12.6%)	0 (0.0%)	
How helpful are your trainers while studying online?	**Extremely helpful**	15 (13.5%)	22 (19.8%)	0.380
	**Very helpful**	44 (39.6%)	40 (36.0%)	
	**Moderately helpful**	42 (37.8%)	37 (33.3%)	
	**Slightly helpful**	9 (8.1%)	12 (10.8%)	
	**Not at all helpful**	1 (0.9%)	0 (0.0%)	
How effective has distance learning been for you?	**Extremely effective**	12 (10.8%)	20 (18.0%)	0.117
	**Very effective**	44 (39.6%)	44 (39.6%)	
	**Moderately effective**	48 (43.2%)	39 (35.1%)	
	**Slightly effective**	6 (5.4%)	8 (7.2%)	
	**Not at all effective**	1 (0.9%)	0 (0.0%)	
How engaged do you feel with the online training process?	**Very engaged**	34 (30.6%)	33 (29.7%)	0.680
	**Somewhat engaged**	35 (31.5%)	40 (36.0%)	
	**Neutral**	37 (33.3%)	34 (30.6%)	
	**Somewhat disengaged**	4 (3.6%)	4 (3.6%)	
	**Very disengaged**	1 (0.9%)	0 (0.0%)	
Overall feeling about distance learning	**Excellent**	21 (18.9%)	33 (29.7%)	0.028*
	**Good**	67 (60.4%)	60 (54.1%)	
	**Average**	21 (18.9%)	18 (16.2%)	
	**Below average**	2 (1.8%)	0 (0.0%)	
LMS is user-friendly and easy to access	**Strongly agree**	0 (0.0%)	40 (36.0%)	0.000*
	**Agree**	62 (55.9%)	43 (38.7%)	
	**Neutral**	46 (41.4%)	28 (25.2%)	
	**Disagree**	2 (1.8%)	0 (0.0%)	
	**Strongly disagree**	1 (0.9%)	0 (0.0%)	
LMS provides comprehensive specialty resources	**Strongly agree**	2 (1.8%)	24 (21.6%)	0.000*
	**Agree**	56 (50.5%)	55 (49.5%)	
	**Neutral**	43 (38.7%)	32 (28.8%)	
	**Disagree**	9 (8.1%)	0 (0.0%)	
	**Strongly disagree**	1 (0.9%)	0 (0.0%)	
LMS resources reflect on clinical & professional development	**Strongly agree**	2 (1.8%)	19 (17.1%)	0.000*
	**Agree**	61 (55.0%)	58 (52.3%)	
	**Neutral**	39 (35.1%)	34 (30.6%)	
	**Disagree**	7 (6.3%)	0 (0.0%)	
	**Strongly disagree**	2 (1.8%)	0 (0.0%)	
LMS helps personalize learning plans	**Strongly agree**	5 (4.5%)	18 (16.2%)	0.001*
	**Agree**	54 (48.6%)	50 (45.0%)	
	**Neutral**	43 (38.7%)	43 (38.7%)	
	**Disagree**	6 (5.4%)	0 (0.0%)	
	**Strongly disagree**	3 (2.7%)	0 (0.0%)	
LMS enables scheduling of foundation courses	**Strongly agree**	7 (6.3%)	17 (15.3%)	0.000*
	**Agree**	50 (45.0%)	57 (51.4%)	
	**Neutral**	42 (37.8%)	37 (33.3%)	
	**Disagree**	10 (9.0%)	0 (0.0%)	
	**Strongly disagree**	2 (1.8%)	0 (0.0%)	
Overall satisfaction with LMS resources	**Strongly satisfied**	3 (2.7%)	24 (21.6%)	0.000*
	**Satisfied**	61 (55.0%)	57 (51.4%)	
	**Neutral**	37 (33.3%)	30 (27.0%)	
	**Dissatisfied**	7 (6.3%)	0 (0.0%)	
	**Strongly dissatisfied**	3 (2.7%)	0 (0.0%)	
Challenges with the LMS	**Accessibility**	28 (25.2%)	28 (25.2%)	0.155
	**Lack of training**	25 (22.5%)	14 (12.6%)	
	**Work overload / time**	17 (15.3%)	9 (8.1%)	
	**Lack of updated resources**	15 (13.5%)	19 (17.1%)	
	**Technical issues**	15 (13.5%)	16 (14.4%)	
	**No challenges**	11 (9.9%)	25 (22.5%)	
LMS increases likelihood of achieving training requirements	**Yes**	68 (61.3%)	97 (87.4%)	0.024*
	**No**	7 (6.3%)	0 (0.0%)	
	**Maybe**	36 (32.4%)	14 (12.6%)	
Areas where LMS had greatest impact	**Knowledge**	58 (52.3%)	47 (42.7%)	0.169
	**Clinical skills**	37 (33.3%)	25 (22.7%)	
	**Foundation courses**	3 (2.7%)	33 (30.0%)	
	**Communication channel**	2 (1.8%)	2 (1.8%)	
	**Uncertain of value**	11 (9.9%)	3 (2.7%)	
Suggestions for improving LMS	**Increase updated resources**	33 (30.0%)	27 (24.8%)	0.099
	**Accessibility/Technical support**	27 (24.5%)	16 (14.7%)	
	**More training/orientation**	20 (18.2%)	27 (24.8%)	
	**Increase foundation courses**	6 (5.5%)	8 (7.3%)	
	**Nothing**	24 (21.8%)	31 (28.4%)	
Additional comments	**More training/orientation**	16 (14.7%)	16 (14.8%)	1.000
	**Frequent updates**	7 (6.4%)	8 (7.4%)	
	**More clinical courses**	7 (6.4%)	4 (3.7%)	
	**Flexible time**	2 (1.8%)	4 (3.7%)	
	**Nothing**	77 (70.6%)	76 (70.4%)	

Data are presented as frequency (%). *LMS* Learning management system. *: significant as *P* value < 0.05

## Discussion

Medical schools had to thoroughly explore reasonable teaching methods to determine how best to teach ‘hands-on’ medicine while protecting students from the deadly contagion. Online education suddenly shifted from a supplementary teaching tool to the primary education mode; this shift included medical education, which further increased educators’ interest. Many educators and scholars feared online education would disrupt traditional classrooms and eventually end on-site schooling. Others argued that online education could not achieve the same outcomes as face-to-face education and should only be used to complement in-person classroom teaching. Evidence-based medicine (EBM) is a compulsory basic course of clinical medicine, preventive medicine, and most other medical specialties in medical colleges [9].

This study demonstrates that structured LMS orientation significantly improves trainees’ perceptions of usability, satisfaction, and perceived educational value. However, it does not significantly influence engagement or contextual barriers related to online learning behaviors.

During the COVID-19 pandemic, medical education underwent a rapid transition from traditional face-to-face instruction to online learning platforms, including in clinical disciplines. This shift raised concerns regarding the effectiveness of virtual education in delivering “hands-on” clinical training, with ongoing debate about whether online learning can fully replace or merely complement conventional teaching methods. Evidence-based medicine (EBM), as a core component of medical curricula, particularly highlights the need for effective integration of digital learning tools into clinical education framework.

In the present study, there were no significant differences in task engagement and device access before and after training. This suggests that although the training program may have enhanced perceptions of the LMS and improved satisfaction levels, it was not sufficient to change existing engagement habits or overcome infrastructural barriers. The consistent preference for small group tasks and the reliance on smartphones and laptops remained unchanged, indicating that learning styles and access limitations are likely influenced by factors beyond short-term educational exposure.

Similarly Rao et al. [10] reported that in Indian postgraduate training settings, technological access and engagement modes were shaped primarily by socioeconomic status and institutional provisions rather than individual-level interventions.

In the present study, no significant differences were found in participants’ experiences with online learning before and after training although most trainees reported feeling comfortable learning at home, common challenges such as distractions, household disturbances, and inconsistent internet connectivity remained prevalent even after the intervention. While there was a slight increase in participants motivated to pursue advanced courses post-training, broader perceptions—including difficulty receiving guidance, social isolation, and limited interactivity—showed no meaningful change.

These findings are consistent with the results of Dost et al. [11], who reported that UK medical students faced persistent environmental distractions and digital fatigue despite transitioning to well-structured online systems during the pandemic.

Similarly, it was found that Pakistani medical students continued to experience home-based disruptions and a lack of conductive study environments, even after being oriented to online platforms [12].

Collectively, these studies reinforce the conclusion that while training can enhance digital literacy, it alone is insufficient to improve the holistic online learning experience, which is deeply influenced by environmental, emotional, and infrastructural factors beyond educational scope.

In the present study, there was no significant difference in participants’ preferred methods for clearing doubts during online learning before and after the training intervention. The majority continued to prefer direct interaction with instructors during or immediately following online sessions, while fewer participants opted to explore additional materials independently or seek peer support. This persistence in preference suggests that despite improved familiarity with the LMS, the training did not substantially shift learners’ reliance on synchronous, instructor-led clarification.

A study by Khalil et al. [13] indicated that while LMS training improved students’ ability to navigate the platform, it did not alter their fundamental preference for live guidance, which they associated with better clarity and trust.

These results suggest that learners in medical and clinical fields may view doubt resolution not merely as a technical or content-based task but as a critical pedagogical interaction that builds confidence and contextual understanding. Therefore, improvements in LMS-based learning environments should focus not only on content delivery but also on integrating real-time, instructor-mediated clarification tools to align with these persistent learner preferences.

In the present study, a significant improvement was observed post-training in participants’ perception of institutional support and their overall attitudes toward distance learning. This suggests that the training program successfully enhanced trainees’ awareness of institutional efforts and possibly increased their trust in the educational infrastructure supporting online learning. However, other critical parameters such as the perceived helpfulness of trainers, learners’ engagement levels, and the overall effectiveness of distance learning—did not demonstrate significant changes. This may indicate that while centralized interventions can influence learners’ general perceptions, more personalized or interactive strategies are needed to improve the educational dynamics between trainers and trainees.

Similar findings were reported by AlQhtani et al. [14], who found that targeted training in LMS use improved students’ appreciation of institutional responsiveness but did not impact perceived instructional quality or engagement in Saudi medical schools.

In the present study, participants demonstrated significant improvements in their perceptions of the Egyptian fellowship LMS following orientation, with notable gains across multiple domains including user-friendliness, system comprehensiveness, clinical reflection, personalization, scheduling efficiency, and overall satisfaction (*p* < .05). These results suggest that targeted LMS training can enhance not only technical navigation but also users’ perception of educational quality and relevance, particularly when aligned with clinical practice needs.

Similarly, Almarzooq et al. [15] found that structured faculty and learner orientation significantly improved perceptions of LMS usability and scheduling flexibility in a postgraduate cardiology program during the COVID-19 pandemic.

In the present study, a significant post-training increase in knowledge was observed in participants’ belief that the LMS played an effective role in helping them meet training requirements and complete foundational course objectives. This finding suggests that structured LMS training can clarify the platform’s alignment with curricular goals and improve user confidence in its role as an academic tool. The improvement likely stems from increased familiarity with features directly related to course planning and outcome tracking.

Likewise, Wilcha [16] emphasized that appropriate training and integration of LMS platforms contributed to improved learner satisfaction with online clinical teaching, particularly when learning outcomes were clearly embedded in the system.

However, in the present study, other domains—including perceived challenges, platform limitations, and suggestions for further improvement—remained statistically unchanged post-training.

This observation aligns with findings from Adnan and Anwar [17], which showed that while training improved familiarity and trust in LMS platforms, it did not necessarily resolve deeper systemic issues such as content gaps, platform responsiveness, or the need for more interactive features. These results underscore that while training interventions can enhance alignment between LMS tools and academic requirements, more extensive revisions in content design, interactivity, and institutional feedback mechanisms may be needed to address persistent concerns and optimize the full learning experience.

The discrepancy between improved perception and unchanged engagement suggests that while trainees better understand the LMS, behavioral change requires addressing external factors such as workload, infrastructure, and learning environment.

We recommended that first, ongoing and comprehensive training sessions based on realistic regular training needs should be implemented for both trainees and trainers to ensure consistent digital competency and effective LMS usage. Second, the LMS content should be regularly updated and tailored to the specific educational and clinical needs of different specialties, enhancing its relevance and applicability. Third, investments in technical infrastructure are essential to ensure equitable access to devices, stable internet connectivity, and platform functionality across all training settings. Additionally, incorporating interactive and engaging tools such as virtual simulations, forums, and real-time assessments could foster deeper engagement and learning outcomes. Finally, a system of continuous monitoring and feedback collection should be established to identify emerging challenges and inform ongoing improvements in digital learning implementation within the Egyptian fellowship program.

Limitations: The study was geographically confined to El-Behera governorate, which may affect the generalizability of the findings to other regions or fellowship centers in Egypt. The follow-up period was relatively short, potentially limiting the assessment of long-term impacts or behavioral changes in LMS engagement. The reliance on self-reported data introduces a risk of response bias, particularly regarding satisfaction and usage patterns. Study did not include in-depth qualitative methods such as interviews or focus groups, which could have provided richer insights into user experiences and contextual challenges. Variability in trainer expertise and LMS engagement was also not controlled for, which may have influenced trainee outcomes. The response rate (52.6%) may introduce selection bias, as more motivated trainees were more likely to complete the post-intervention assessment. The absence of a control group limits causal inference. Additionally, reliance on self-reported data may overestimate perceived improvements.

## Conclusions

LMS-based orientation improves trainees’ perceptions but is insufficient to enhance engagement or overcome systemic barriers. Future strategies should focus on interactive design, continuous training, and infrastructure support to optimize LMS integration in postgraduate medical education.

## Supplementary Information

Supplementary Material 1.

## Data Availability

The datasets used and/or analyzed during the current study are available from the corresponding author on reasonable request.

## References

[1] Yoong SQ, Liao AWX, Goh SH, Zhang H. Educational effects of community service-learning involving older adults in nursing education: An integrative review. Nurse Educ Today. 2022;113:105376. 35489329 10.1016/j.nedt.2022.105376

[2] Nassar MFMA. Continuing professional development in the healthcare sector in Egypt: a readiness assessment. 2018

[3] Mortagy M, Abdelhameed A, Sexton P, Olken M, Hegazy MT, Gawad MA, et al. Online medical education in Egypt during the COVID-19 pandemic: A nationwide assessment of medical students’ usage and perceptions. BMC Med Educ. 2022;22:218. 35354406 10.1186/s12909-022-03249-2 PMC8966850

[4] Ismail HA, Hafiz M, Soliman R. Sustainability and resilience in the Egyptian health system. LSE; 2024.

[5] Doron R, Eichler R, Rajhans V. Effectiveness of online learning in improving optometry student’s reflective abilities. J Optom. 2023;16:199–205. 36400680 10.1016/j.optom.2022.10.001 PMC9666351

[6] Darius PSH, Gundabattini E, Solomon DG. A survey on the effectiveness of online teaching–learning methods for university and college students. J Institution Eng (India): Ser B. 2021;102:1325–34.

[7] Bhat A. Distance learning survey for students: Tips & examples. 2023. Retrieved from https://www.questionpro.com/blog/distance-learning-survey-questions-for-students/.

[8] Prabu Kumar A, Omprakash A, Chokkalingam Mani PK, Kuppusamy M, Wael D, Sathiyasekaran BWC, et al. E-learning and E-modules in medical education-A SOAR analysis using perception of undergraduate students. PLoS ONE. 2023;18:e0284882. 37205679 10.1371/journal.pone.0284882 PMC10198563

[9] Babu R, Bahuleyan B, Panchu P, Shilpa A, Sreeja C, Manjuran DJ. Effectiveness of self-paced and instructor-led online learning: a study among Phase I medical students. J Clin Diagn Res. 2022;16:1.

[10] Rao LN, Shetty A, Pai V, Natarajan S, Baliga MS, Wahjuningrum DA, et al. Perceptions and challenges of online teaching and learning amidst the COVID-19 pandemic in India: A cross-sectional study with dental students and teachers. BMC Med Educ. 2024;24:637. 38844924 10.1186/s12909-024-05340-2 PMC11157739

[11] Dost S, Hossain A, Shehab M, Abdelwahed A, Al-Nusair L. Perceptions of medical students towards online teaching during the COVID-19 pandemic: A national cross-sectional survey of 2721 UK medical students. BMJ Open. 2020;10:e042378. 10.1136/bmjopen-2020-042378 PMC7646323 33154063

[12] Elumalai KV, Sankar JP, Kalaichelvi R, John JA, Menon N, Alqahtani MSM, et al. Factors affecting the quality of e-learning during the COVID-19 pandemic from the perspective of higher education students. COVID-19 Education: Learn Teach Pandemic-Constrained Environ. 2021;189:169.

[13] Khalil R, Mansour AE, Fadda WA, Almisnid K, Aldamegh M, Al-Nafeesah A, et al. The sudden transition to synchronized online learning during the COVID-19 pandemic in Saudi Arabia: A qualitative study exploring medical students’ perspectives. BMC Med Educ. 2020;20:285. 32859188 10.1186/s12909-020-02208-z PMC7453686

[14] AlQhtani A, AlSwedan N, Almulhim A, Aladwan R, Alessa Y, AlQhtani K, et al. Online versus classroom teaching for medical students during COVID-19: Measuring effectiveness and satisfaction. BMC Med Educ. 2021;21:452. 34454493 10.1186/s12909-021-02888-1 PMC8397601

[15] Almarzooq ZI, Lopes M, Kochar A. Virtual learning during the COVID-19 pandemic: A disruptive technology in graduate medical education. Am Coll Cardiol Foundation Wash DC. 2020;75:2635–8. 10.1016/j.jacc.2020.04.015 PMC7159871 32304797

[16] Wilcha R-J. Effectiveness of virtual medical teaching during the COVID-19 crisis: Systematic review. JMIR Med Educ. 2020;6:e20963. 33106227 10.2196/20963 PMC7682786

[17] Adnan M, Anwar K. Online learning amid the COVID-19 pandemic: Students’ perspectives. Online Submiss. 2020;2:45–51.

