# Supplementary material for: Learning management system effectiveness and trainee perceptions
Source: BMC Med Educ. 2026 Jun 4;26:911. doi: 10.1186/s12909-026-09535-7 (PMC13235011; doi:10.1186/s12909-026-09535-7)
Supplement: Supplementary file 1 — Supplementary Material 1. [file 12909_2026_9535_MOESM1_ESM.pdf]

Detecting the challenges of using E-learning and learning management system platform at Egyptian fellowship training, and the ways of improvement

استبيان لرصد تحديات التعامل مع منصات التعليم الإليكترونى بالزمالة المصرية و طرق التحسين

mostafa.sa.madboly@gmail.com Switch account

Not shared

* Indicates required question

Egyptian Fellowship LMS
mohp-hcms.ekb.eg

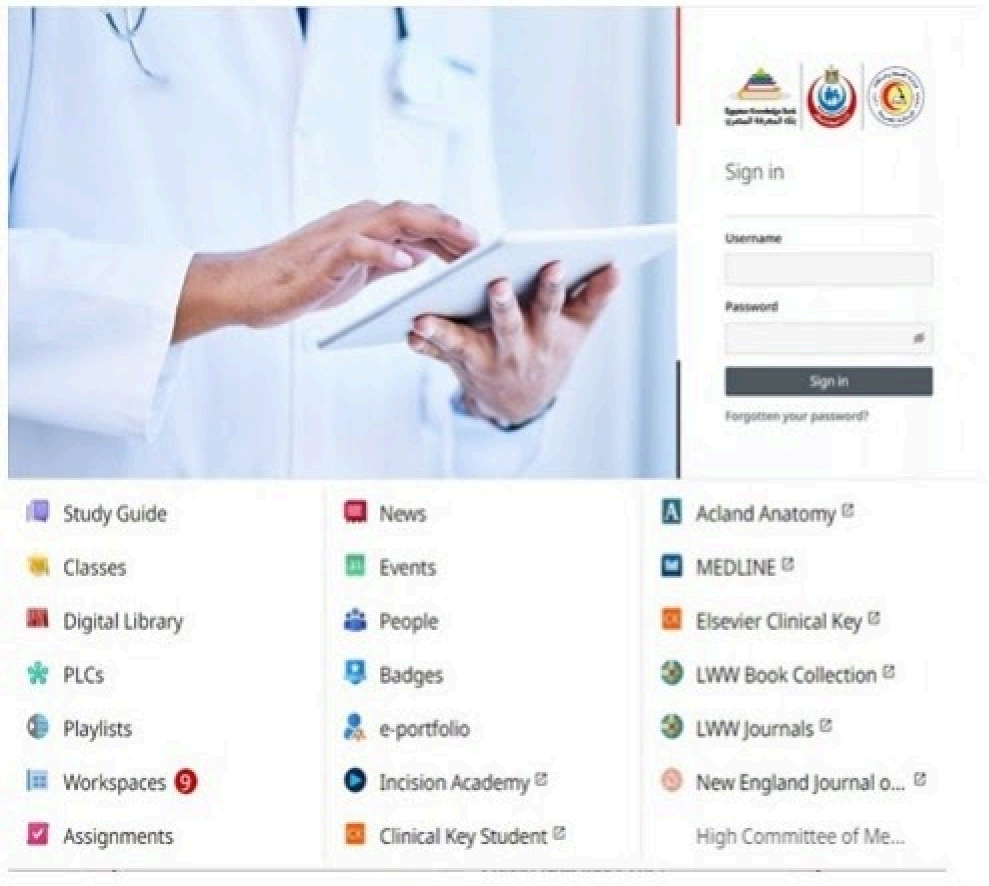

1- In this survey we are trying to detect the challenges facing you in online learning in general and that of the Egyptian fellowship LMS and to find the possible ways of improvement, do you agree to participate in this research survey? *

- Yes
- No

2-Age *

Your answer

3-Gender *

- ◯ Male
- ◯ Female

4-Specialty *

Choose ▾

5-Training center/ hospital *

Choose ▾

6- Which of the methods engaged & enabled you personally to work on a specific task at ease? *

- ☐ Individual assignment
- ☐ Small group task (less than 10 college)
- ☐ Large group task (10 or more college)

7- Do you have access to a device for learning online? *

- ◯ Yes, 100 % of time available
- ◯ less than 50% of time available
- ◯ No, I share with others

8- Which of the following devices do you use for your online learning? *

- ☐ Laptop
- ☐ Desktop computer
- ☐ Tablet
- ☐ Smartphone

9-Your experience with online learning at home *

- ☐ I am learning at my own place comfortably
- ☐ My situational challenges are not suitable
- ☐ I can learn better with uninterrupted network connectivity
- ☐ I am distracted with various activities at home
- ☐ I have many responsibilities, I do not have any time left for online learning

10- Which the following is true regarding online learning *

- ◯ No one disturb me during online learning
- ◯ My family member /roommate occasionally disturb me during online learning
- ◯ My family member /roommate constantly disturb me during online learning

11- Regarding your interest in computer and adoption of online learning, which of these is true? *

- It is difficult to adopt online learning without appropriate guidance
- It is difficult to favor online learning on regular basis due to least face to face interactions
- Slow computers and poor internet connections discourage online learning
- Online learning is often avoided as it promotes social isolation
- Online learning highly motivates the students for taking advanced courses

12-?Did you receive training about E-learning platforms *

- Yes
- No

13- ?Who trained you about it *

- your scientific supervisor
- your trainer
- your senior trainee
- self directed

14- What is your preferred method for clearing doubts in online learning? *

- Ask the professor during / after the online session
- Go through online material providing additional resources
- Post the query to get help from your class peers
- Other:

15- How helpful your University has been in offering you the resources to learn from home? *

- Extremely helpful
- Very helpful
- Moderately helpful
- Slightly helpful
- Not at all helpful

16- How helpful are your trainers while studying online? *

- Extremely helpful
- Very helpful
- Moderately helpful
- Slightly helpful
- Not at all helpful

17- How effective has distance learning been for you? *

- Extremely effective
- Very effective
- Moderately effective
- Slightly effective
- Not at all effective

18- How engaged do you feel with the online training process? *

- Very engaged
- Somewhat engaged
- Neutral
- Somewhat disengaged
- Very disengaged

19- How do you feel overall about distance learning? *

- Excellent
- Good
- Average
- Below average
- Poor

please answer the following questions about Egyptian LMS platform

On a scale of 1 to 5, with 1 being strongly agree and 5 being strongly disagree, please rate your agreement with the following statements

20- Egyptian fellowship LMS is used friendly and you can access its libraries and playlists easily *

- 1-Strongly agree
- 2-Agree
- 3-Neutral
- 4- Disagree
- 5- Strongly disagree

21- Egyptian fellowship LMS provides comprehensive resources and materials in your specialty (knowledge - skills - attitude) *

- 1-Strongly agree
- 2-Agree
- 3-Neutral
- 4- Disagree
- 5- Strongly disagree

22- Egyptian fellowship LMS resources reflected on your clinical experiences & professional development *

- 1-Strongly agree
- 2-Agree
- 3-Neutral
- 4- Disagree
- 5- Strongly disagree

23- Egyptian fellowship LMS resources help you to personalize your learning plan and focus on areas of interest *

- 1-Strongly agree
- 2-Agree
- 3-Neutral
- 4- Disagree
- 5- Strongly disagree

24- Egyptian fellowship LMS enables you to schedule your foundation courses effectively *

- 1-Strongly agree
- 2-Agree
- 3-Neutral
- 4- Disagree
- 5- Strongly disagree

25- In general, you are satisfied with the resources provided on Egyptian fellowship LMS *

- 1-Strongly agree
- 2-Agree
- 3-Neutral
- 4- Disagree
- 5- Strongly disagree

26- From your experience, what are the challenges or concerns regarding Egyptian fellowship LMS in your fellowship training? *

Your answer

27- Do you believe that the Egyptian fellowship LMS has positively increase your likelihood of achieving your training requirements and foundation courses needed?- *

- Yes
- No
- Maybe

28- In what areas of your clinical practice do you feel that Egyptian fellowship LMS had the greatest impact *

Your answer

29- What are your **suggestions** for the improvement of the Egyptian fellowship LMS *

Your answer

30- Please provide any additional comments or feedback regarding Egyptian fellowship LMS *

Your answer

Best wishes

Submit

Clear form

Never submit passwords through Google Forms.

This content is neither created nor endorsed by Google. - Contact form owner - Terms of Service - Privacy Policy

Does this form look suspicious? Report

Google Forms
